# The complete chloroplast genome of *Hemerocallis citrina* (Asphodelaceae), an ornamental and medicinal plant

**DOI:** 10.1080/23802359.2020.1726227

**Published:** 2020-02-11

**Authors:** Xiaobin Ou, Ge Liu, Li-Hong Wu

**Affiliations:** a College of Life Sciences & Technology, Longdong University, Gansu, China;; b Nanjing University of Aeronautics and Astronautics University, Nanjing, China;; c College of Life Sciences, Zhejiang University, Hangzhou, China

**Keywords:** *Hemerocallis citrin*, chloroplast genome, Asphodelaceae

## Abstract

*Hemerocallis citrina* (Asphodelaceae) has been wildly cultivated as ornamental and medicinal plant. Here, we reported the first chloroplast genome sequence of *H. citrina*. The chloroplast genome size is 156,088 bp with GC content of 37.3%, including a large single-copy (LSC) of 84,843 bp, a small single-copy (SSC) of 18,507 bp, and a pair of 26,369 bp IR(inverted repeat) regions. A total of 133 genes were annotated including 87 protein-coding genes, 38 tRNA genes, and 8 rRNA genes. The phylogenetic analysis revealed that *H. citrina* belongs to the *Hemerocallis* genus in Asphodelaceae family.

*Hemerocallis citrina* Baroni., common names Citron daylily and long yellow daylily, is a species of herbaceous perennial plant in the family Asphodelaceae, which is native to central and northern China, the Korea Peninsula, and Japan (Hou et al. 2017). Citron dayliy is now cultivated widely in Asia as ornamental plant and vegetable plant because of its beautiful flower, pleasant flavor, and beneficial secondary metabolites (Lin et al. 2013). In addition, It has been used for medicinal purposes such as relieving gloom and improving sleeping (Yang et al. 2017). Despite its great ornamental and medical importance, there are a few chloroplast markers for breeding of this species. In this study, we sequenced and assembled the complete chloroplast genome sequence of *H. citrina* and reconstructed the phylogenetic relationship with other Asphodelaceae species. Such a plastome sequence could provide abundant genetic information for identification, utilization, and breeding of this species.

The leaves of *H. citrina* was collected from Qingyang, Gansu, China (N35°43′47.2″ E107°42′1.3″). The voucher specimen was deposited in the Hebarium of Longdong University, Gansu, China (Accession NO. XB20190913). Genomic DNA was extracted using a standard CTAB method (Murray & Thompson 1980). Sequencing was conducted on HiSeq^TM^2500 (Illumina, San Diego, California, USA) with 150 bp paired-end sequencing. The complete plastome sequence was constructed using GetOrganelle (Jin et al. 2018) and annotated using Geneious Prime 2019.1.1 (www.geneious.com) by comparing with the plastome of *Hemerocallis fulva* (GenBank Accession No. MG914655), followed by manual inspection. The new annotated chloroplast sequences were deposited in GenBank (MN872235).

The complete chloroplast genome of *H. citrin* was 156,099 bp length with a GC content of 37.3%. It consists of a pair of IR (inverted repeat) regions of 26,369 bp, separated by a 84,843 bp LSC (large single-copy) and a 18,507 bp SSC (small single-copy) regions. A total of 133 genes were annotated, including 87 protein-coding genes, 38 tRNA genes, and 8 rRNA genes, and the IR regions contain 20 duplicate genes.

In order to identify systematic position of *H. citrina,* we conducted a phylogenetic analysis using whole chloroplast genomes of *H. citrina* and the other reported 11 related species with *Cymbidium faberi* as an outgroup. The sequences were aligned using MAFFT 7.017 (Nakamura et al. 2018). The best-fitting model of nucleotide substitution was GTR + G, as determined by the Akaike Information Criterion (AIC) in jModelTest v. 2.1.7 (Darriba et al. 2012). ML (maximum likelihood) analysis was conducted using RAxML- HPC v. 8.2.8 with 1000 bootstrap replicates on the CIPRES Science Gateway website (Miller et al. 2010). Phylogenetic result strongly supported *H. citrina* belongs to the *Hemerocallis* genus in Asphodelaceae family (Figure 1).

**Figure 1.**
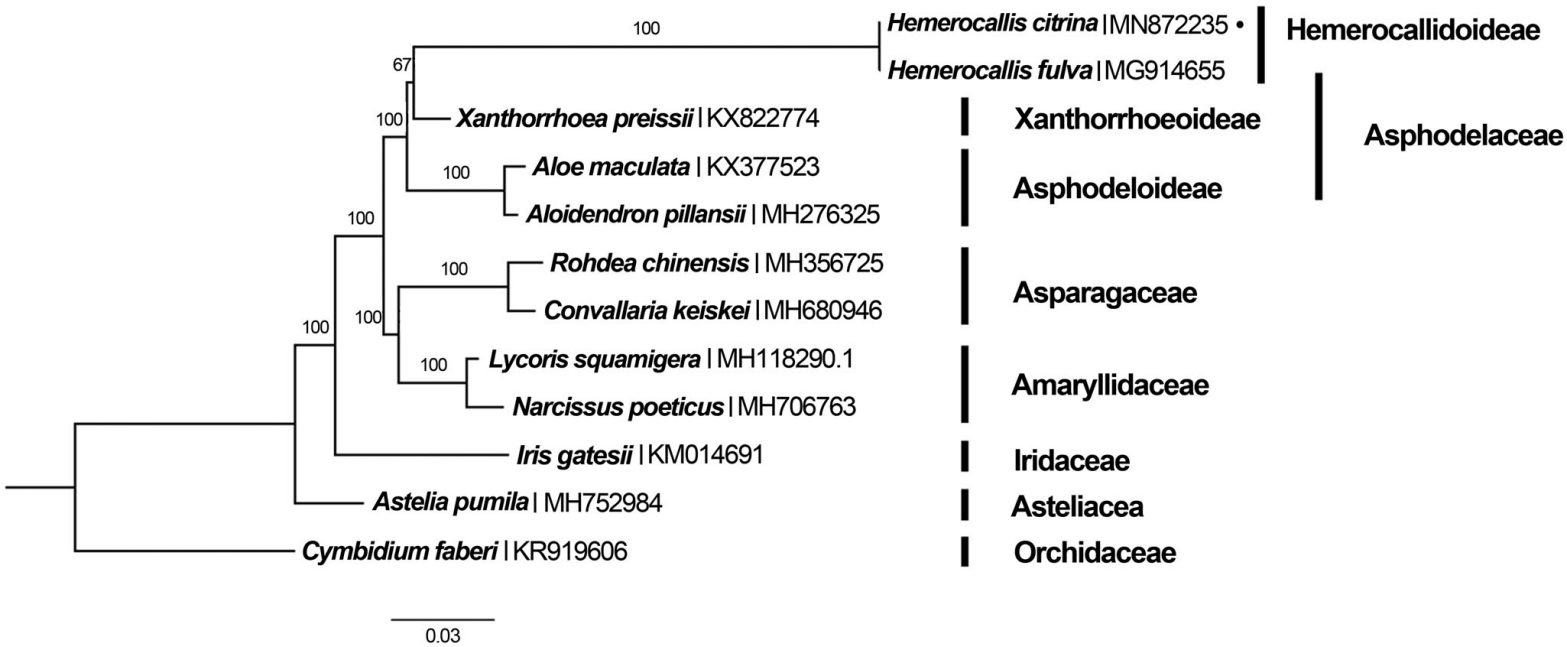
Phylogenetic tree using maximum likelihood (ML) based on plastomes of 11 related species and 1 outgroups with 1000 bootstrap replicates. Relative branch lengths are indicated. Numbers near the nodes represent ML bootstrap values.
